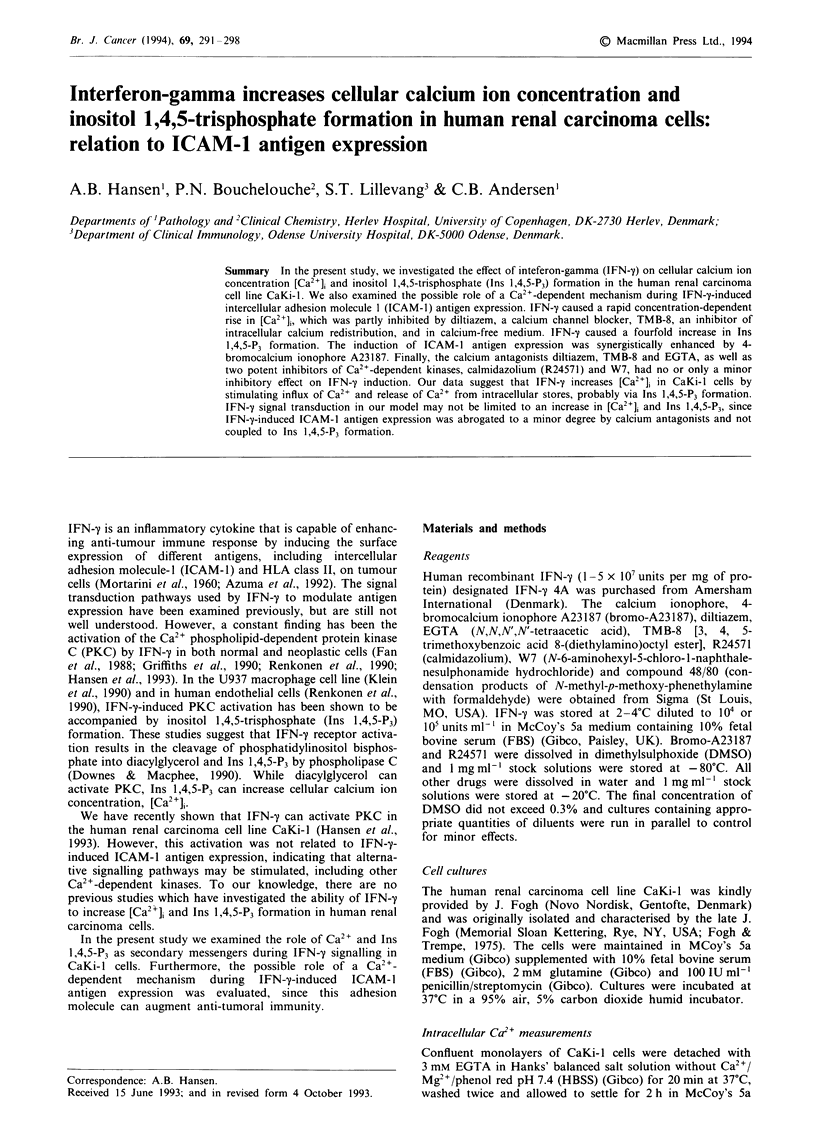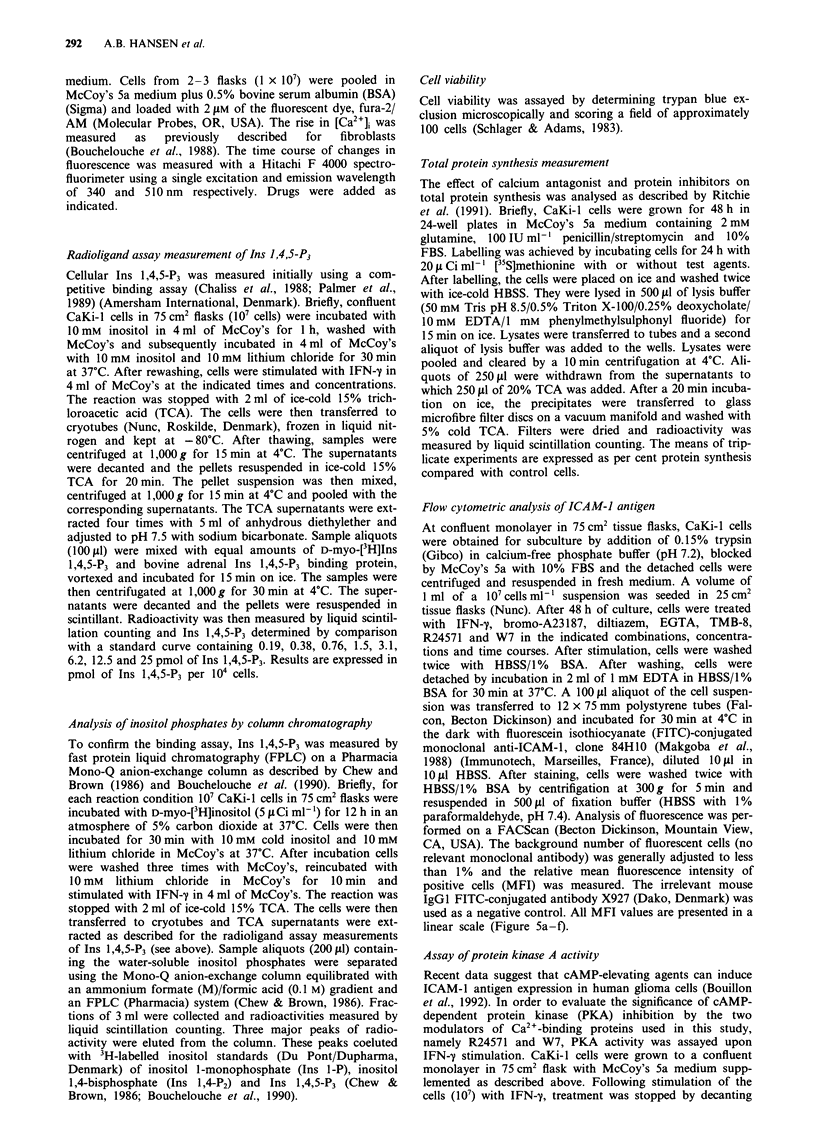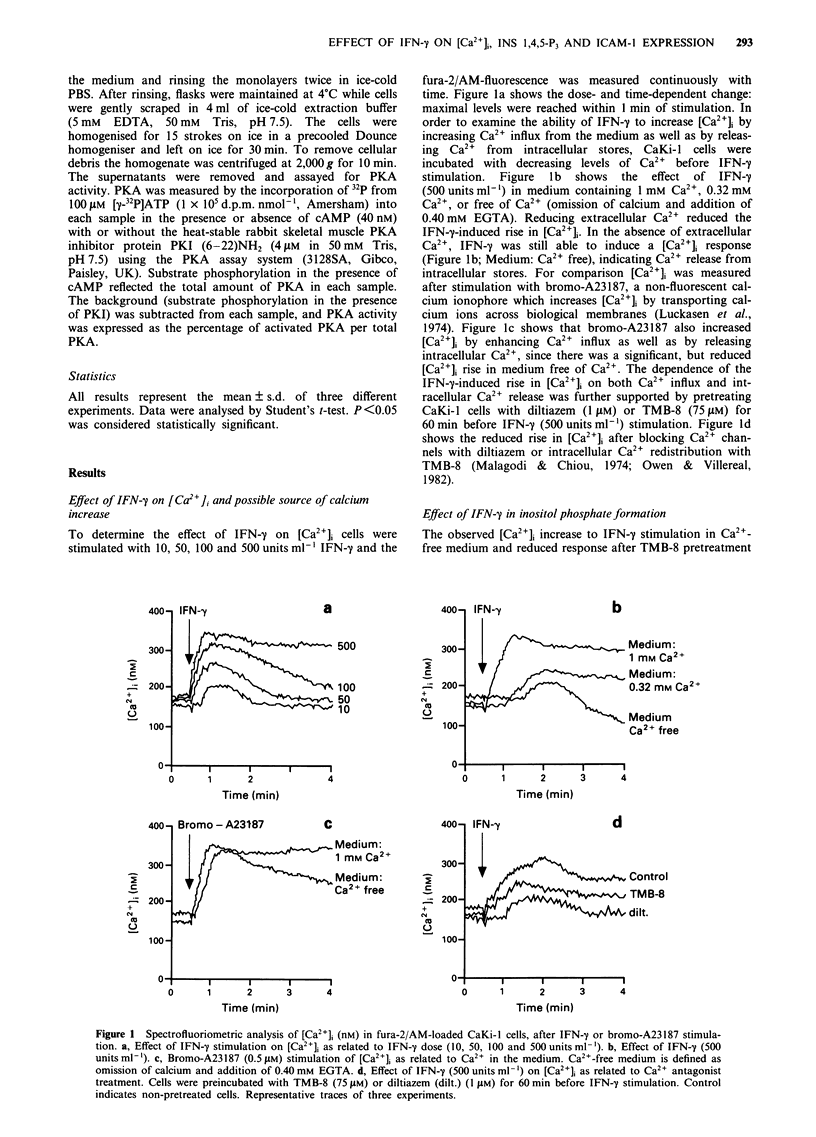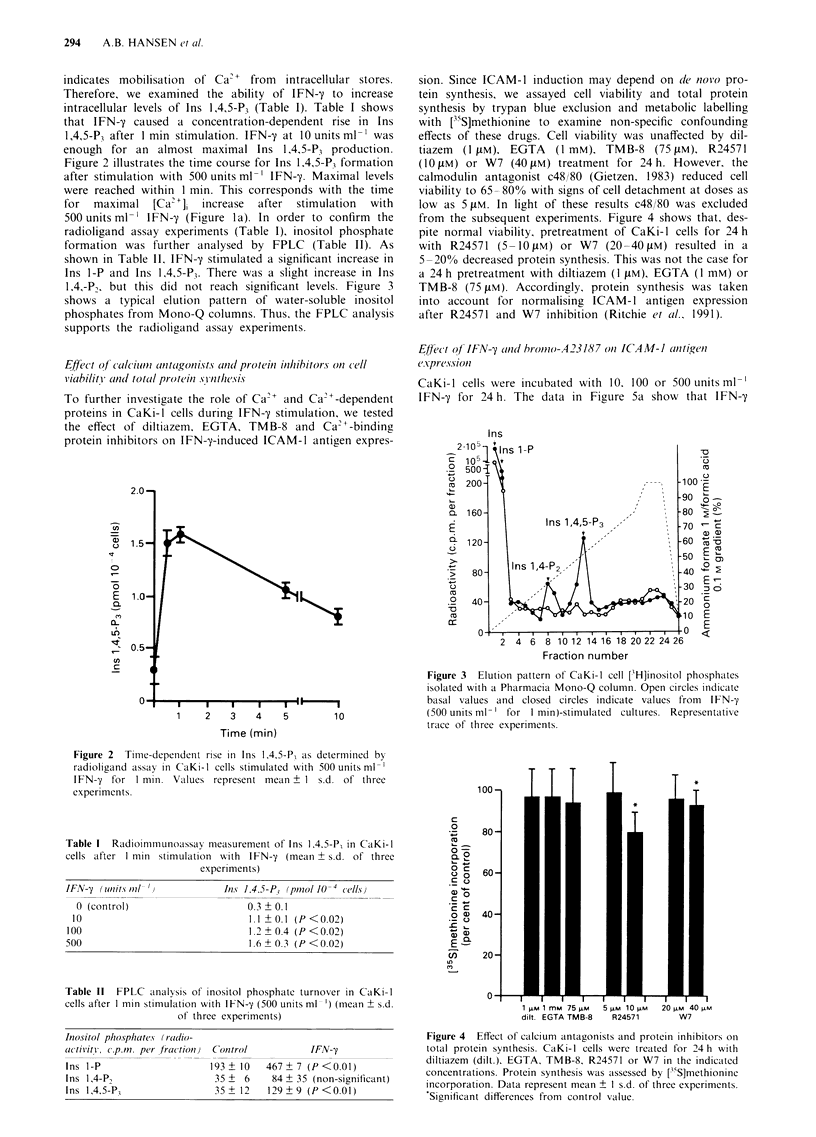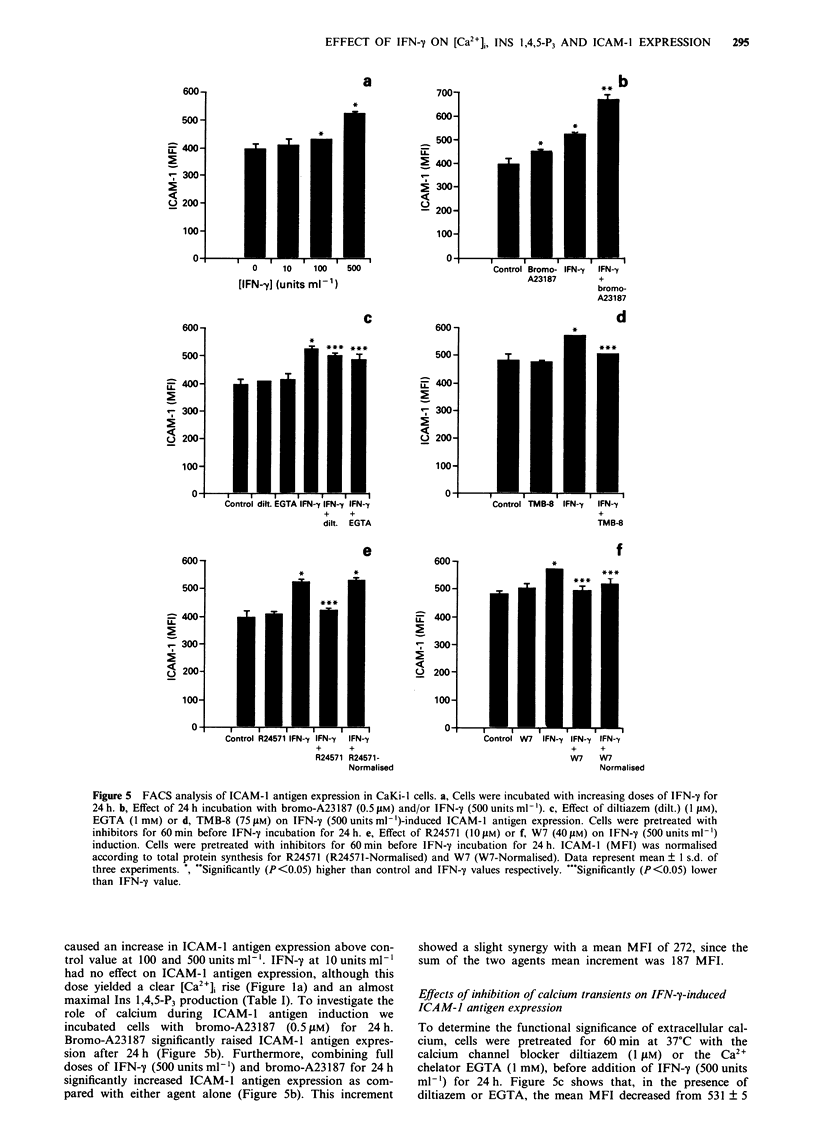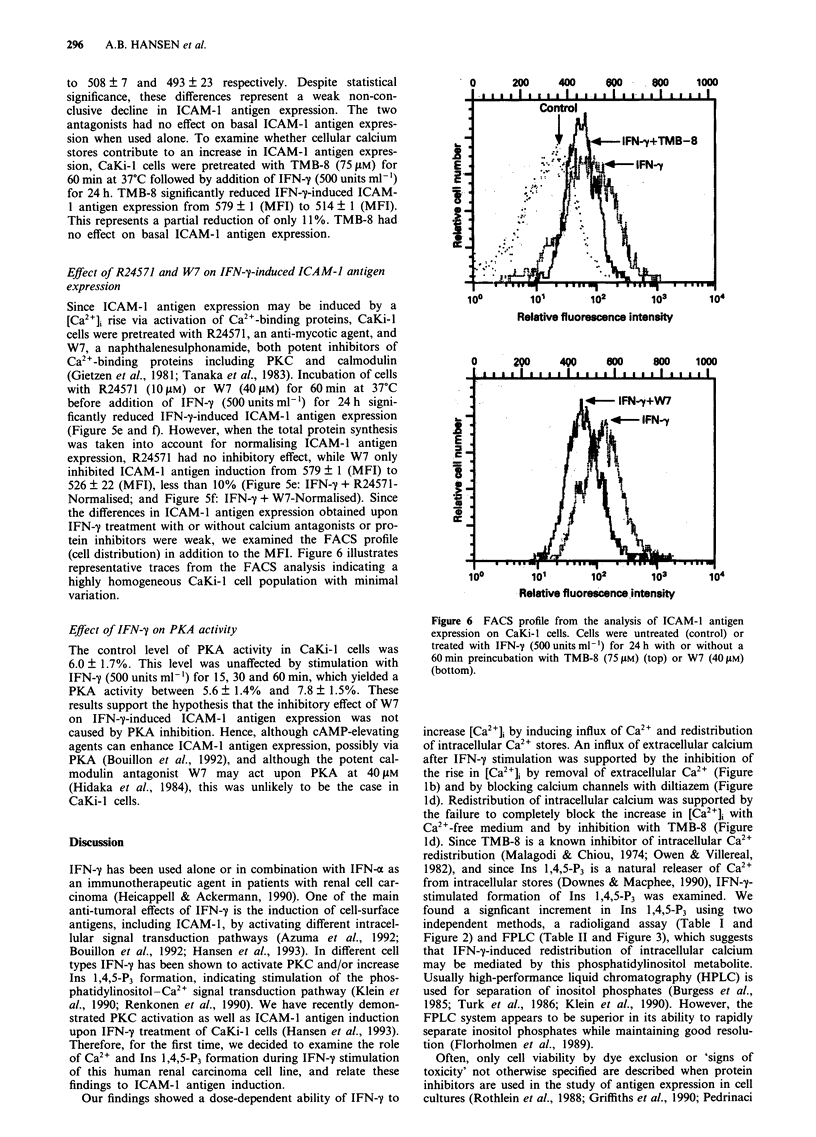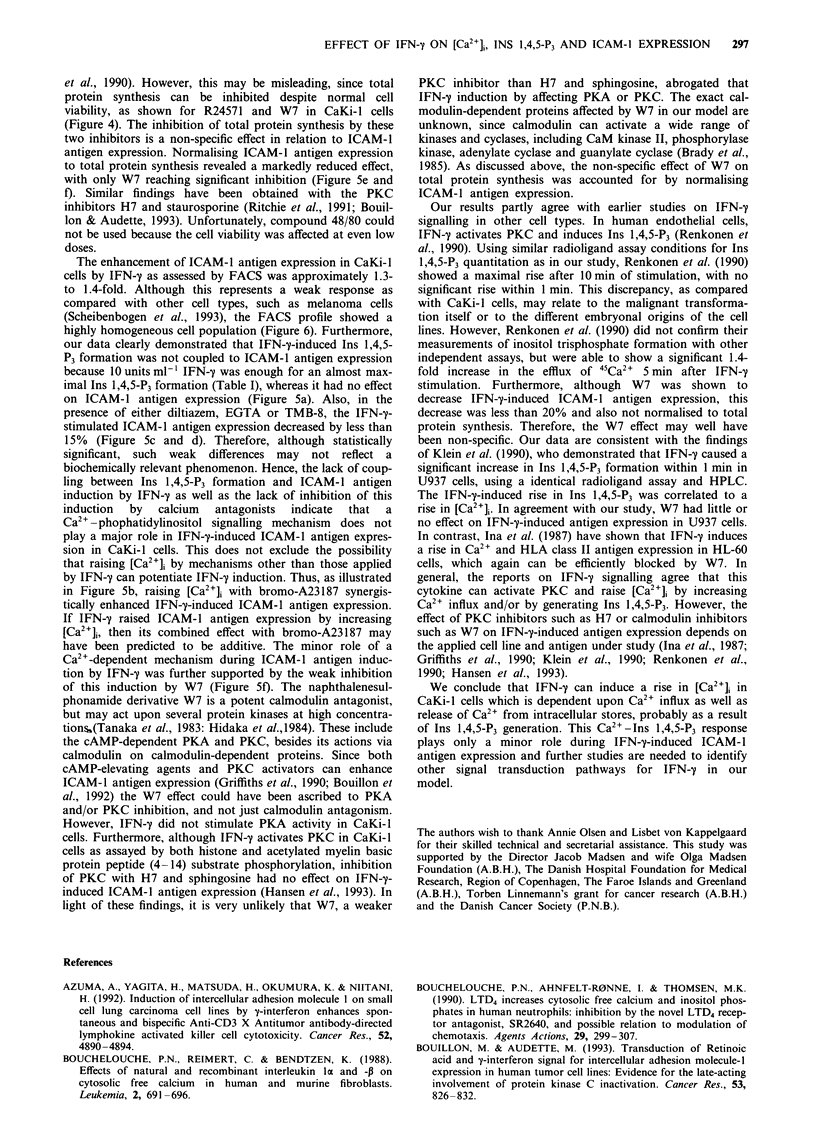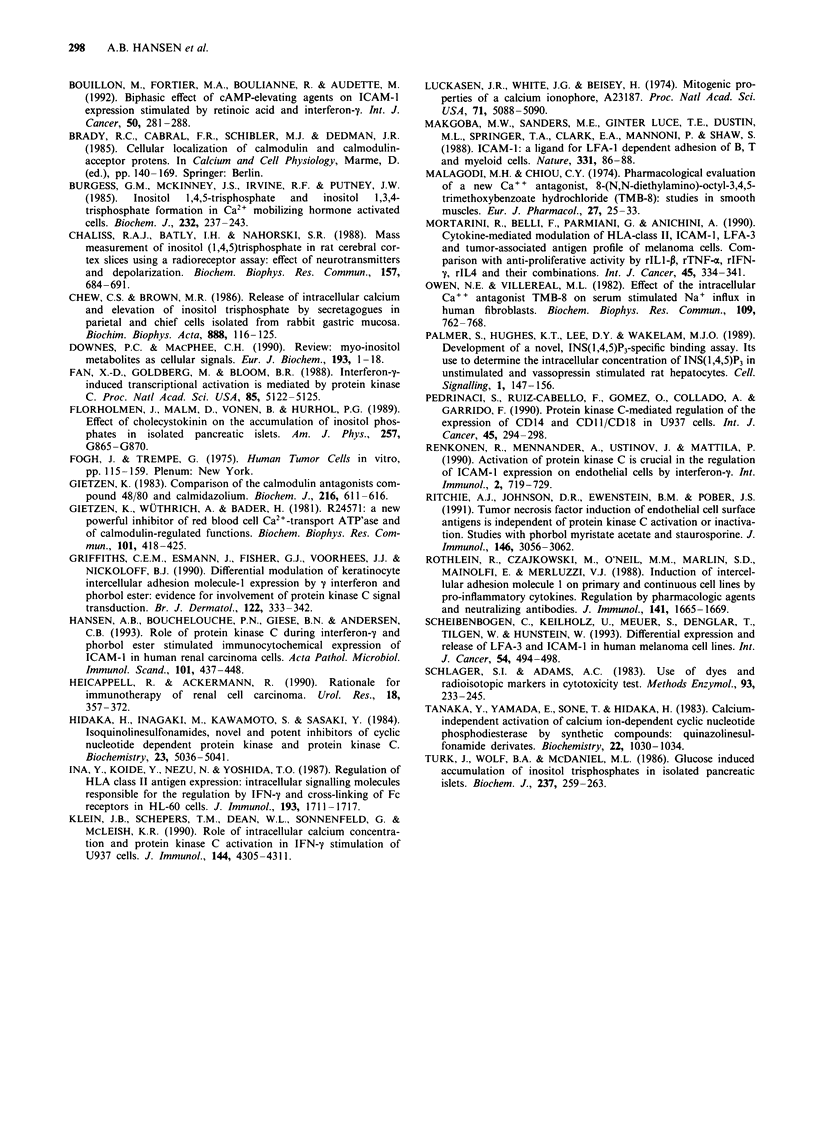# Interferon-gamma increases cellular calcium ion concentration and inositol 1,4,5-trisphosphate formation in human renal carcinoma cells: relation to ICAM-1 antigen expression.

**DOI:** 10.1038/bjc.1994.54

**Published:** 1994-02

**Authors:** A. B. Hansen, P. N. Bouchelouche, S. T. Lillevang, C. B. Andersen

**Affiliations:** Department of Pathology, Herlev Hospital, University of Copenhagen, Denmark.

## Abstract

In the present study, we investigated the effect of interferon-gamma (IFN-gamma) on cellular calcium ion concentration [Ca2+]i and inositol 1,4,5-trisphosphate (Ins 1,4,5-P3) formation in the human renal carcinoma cell line CaKi-1. We also examined the possible role of a Ca(2+)-dependent mechanism during IFN-gamma-induced intercellular adhesion molecule 1 (ICAM-1) antigen expression. IFN-gamma caused a rapid concentration-dependent rise in [Ca2+]i, which was partly inhibited by diltiazem, a calcium channel blocker, TMB-8, an inhibitor of intracellular calcium redistribution, and in calcium-free medium. IFN-gamma caused a fourfold increase in Ins 1,4,5-P3 formation. The induction of ICAM-1 antigen expression was synergistically enhanced by 4-bromocalcium ionophore A23187. Finally, the calcium antagonists diltiazem. TMB-8 and EGTA, as well as two potent inhibitors of Ca(2+)-dependent kinases, calmidazolium (R24571) and W7, had no or only a minor inhibitory effect on IFN-gamma induction. Our data suggest that IFN-gamma increases [Ca2+]i in CaKi-1 cells by stimulating influx of Ca2+ and release of Ca2+ from intracellular stores, probably via Ins 1,4,5-P3 formation. IFN-gamma signal transduction in our model may not be limited to an increase in [Ca2+]i and Ins 1,4,5-P3, since IFN-gamma-induced ICAM-1 antigen expression was abrogated to a minor degree by calcium antagonists and not coupled to Ins 1,4,5-P3 formation.


Br. J. Cancer (1994), 69, 291–298 © Macmillan Press Ltd., 1994

### Interferon-gamma increases cellular calcium ion concentration and inositol 1,4,5-trisphosphate formation in human renal carcinoma cells: relation to ICAM-1 antigen expression

A.B. Hansen[1], P.N. Bouchelouche[2], S.T. Lillevang[3] & C.B. Andersen[1]

*Departments of [1]Pathology and [2]Clinical Chemistry, Herlev Hospital, University of Copenhagen, DK-2730 Herlev, Denmark; [3]Department of Clinical Immunology, Odense University Hospital, DK-5000 Odense, Denmark.*

**Summary** In the present study, we investigated the effect of inteferon-gamma (IFN-γ) on cellular calcium ion concentration $[Ca^{2+}]_i$ and inositol 1,4,5-trisphosphate (Ins 1,4,5-$P_3$) formation in the human renal carcinoma cell line CaKi-1. We also examined the possible role of a $Ca^{2+}$-dependent mechanism during IFN-γ-induced intercellular adhesion molecule 1 (ICAM-1) antigen expression. IFN-γ caused a rapid concentration-dependent rise in $[Ca^{2+}]_i$, which was partly inhibited by diltiazem, a calcium channel blocker, TMB-8, an inhibitor of intracellular calcium redistribution, and in calcium-free medium. IFN-γ caused a fourfold increase in Ins 1,4,5-$P_3$ formation. The induction of ICAM-1 antigen expression was synergistically enhanced by 4-bromocalcium ionophore A23187. Finally, the calcium antagonists diltiazem, TMB-8 and EGTA, as well as two potent inhibitors of $Ca^{2+}$-dependent kinases, calmidazolium (R24571) and W7, had no or only a minor inhibitory effect on IFN-γ induction. Our data suggest that IFN-γ increases $[Ca^{2+}]_i$ in CaKi-1 cells by stimulating influx of $Ca^{2+}$ and release of $Ca^{2+}$ from intracellular stores, probably via Ins 1,4,5-$P_3$ formation. IFN-γ signal transduction in our model may not be limited to an increase in $[Ca^{2+}]_i$ and Ins 1,4,5-$P_3$, since IFN-γ-induced ICAM-1 antigen expression was abrogated to a minor degree by calcium antagonists and not coupled to Ins 1,4,5-$P_3$ formation.

IFN-γ is an inflammatory cytokine that is capable of enhancing anti-tumour immune response by inducing the surface expression of different antigens, including intercellular adhesion molecule-1 (ICAM-1) and HLA class II, on tumour cells (Mortarini *et al.*, 1960; Azuma *et al.*, 1992). The signal transduction pathways used by IFN-γ to modulate antigen expression have been examined previously, but are still not well understood. However, a constant finding has been the activation of the $Ca^{2+}$ phospholipid-dependent protein kinase C (PKC) by IFN-γ in both normal and neoplastic cells (Fan *et al.*, 1988; Griffiths *et al.*, 1990; Renkonen *et al.*, 1990; Hansen *et al.*, 1993). In the U937 macrophage cell line (Klein *et al.*, 1990) and in human endothelial cells (Renkonen *et al.*, 1990), IFN-γ-induced PKC activation has been shown to be accompanied by inositol 1,4,5-trisphosphate (Ins 1,4,5-$P_3$) formation. These studies suggest that IFN-γ receptor activation results in the cleavage of phosphatidylinositol bisphosphate into diacylglycerol and Ins 1,4,5-$P_3$ by phospholipase C (Downes & Macphee, 1990). While diacylglycerol can activate PKC, Ins 1,4,5-$P_3$ can increase cellular calcium ion concentration, $[Ca^{2+}]_i$.

We have recently shown that IFN-γ can activate PKC in the human renal carcinoma cell line CaKi-1 (Hansen *et al.*, 1993). However, this activation was not related to IFN-γ-induced ICAM-1 antigen expression, indicating that alternative signalling pathways may be stimulated, including other $Ca^{2+}$-dependent kinases. To our knowledge, there are no previous studies which have investigated the ability of IFN-γ to increase $[Ca^{2+}]_i$ and Ins 1,4,5-$P_3$ formation in human renal carcinoma cells.

In the present study we examined the role of $Ca^{2+}$ and Ins 1,4,5-$P_3$ as secondary messengers during IFN-γ signalling in CaKi-1 cells. Furthermore, the possible role of a $Ca^{2+}$-dependent mechanism during IFN-γ-induced ICAM-1 antigen expression was evaluated, since this adhesion molecule can augment anti-tumoral immunity.

Correspondence: A.B. Hansen.
Received 15 June 1993; and in revised form 4 October 1993.

### Materials and methods

### *Reagents*

Human recombinant IFN-γ (1–5 × $10^7$ units per mg of protein) designated IFN-γ 4A was purchased from Amersham International (Denmark). The calcium ionophore, 4-bromocalcium ionophore A23187 (bromo-A23187), diltiazem, EGTA (*N,N,N′,N′*-tetraacetic acid), TMB-8 [3, 4, 5-trimethoxybenzoic acid 8-(diethylamino)octyl ester], R24571 (calmidazolium), W7 (*N*-6-aminohexyl-5-chloro-1-naphthalenesulphonamide hydrochloride) and compound 48/80 (condensation products of *N*-methyl-*p*-methoxy-phenethylamine with formaldehyde) were obtained from Sigma (St Louis, MO, USA). IFN-γ was stored at 2–4°C diluted to $10^4$ or $10^5$ units $ml^{-1}$ in McCoy's 5a medium containing 10% fetal bovine serum (FBS) (Gibco, Paisley, UK). Bromo-A23187 and R24571 were dissolved in dimethylsulphoxide (DMSO) and 1 mg $ml^{-1}$ stock solutions were stored at −80°C. All other drugs were dissolved in water and 1 mg $ml^{-1}$ stock solutions were stored at −20°C. The final concentration of DMSO did not exceed 0.3% and cultures containing appropriate quantities of diluents were run in parallel to control for minor effects.

### *Cell cultures*

The human renal carcinoma cell line CaKi-1 was kindly provided by J. Fogh (Novo Nordisk, Gentofte, Denmark) and was originally isolated and characterised by the late J. Fogh (Memorial Sloan Kettering, Rye, NY, USA; Fogh & Trempe, 1975). The cells were maintained in MCoy's 5a medium (Gibco) supplemented with 10% fetal bovine serum (FBS) (Gibco), 2 mM glutamine (Gibco) and 100 IU $ml^{-1}$ penicillin/streptomycin (Gibco). Cultures were incubated at 37°C in a 95% air, 5% carbon dioxide humid incubator.

### *Intracellular $Ca^{2+}$ measurements*

Confluent monolayers of CaKi-1 cells were detached with 3 mM EGTA in Hanks' balanced salt solution without $Ca^{2+}$/$Mg^{2+}$/phenol red pH 7.4 (HBSS) (Gibco) for 20 min at 37°C, washed twice and allowed to settle for 2 h in McCoy's 5a medium. Cells from 2–3 flasks ($1 \times 10^7$) were pooled in McCoy's 5a medium plus 0.5% bovine serum albumin (BSA) (Sigma) and loaded with 2 μM of the fluorescent dye, fura-2/AM (Molecular Probes, OR, USA). The rise in $[Ca^{2+}]_i$ was measured as previously described for fibroblasts (Bouchelouche et al., 1988). The time course of changes in fluorescence was measured with a Hitachi F 4000 spectrofluorimeter using a single excitation and emission wavelength of 340 and 510 nm respectively. Drugs were added as indicated.

*Radioligand assay measurement of Ins 1,4,5-P$_3$*

Cellular Ins 1,4,5-$P_3$ was measured initially using a competitive binding assay (Chaliss et al., 1988; Palmer et al., 1989) (Amersham International, Denmark). Briefly, confluent CaKi-1 cells in 75 cm$^2$ flasks ($10^7$ cells) were incubated with 10 mM inositol in 4 ml of McCoy's for 1 h, washed with McCoy's and subsequently incubated in 4 ml of McCoy's with 10 mM inositol and 10 mM lithium chloride for 30 min at 37°C. After rewashing, cells were stimulated with IFN-γ in 4 ml of McCoy's at the indicated times and concentrations. The reaction was stopped with 2 ml of ice-cold 15% trichloroacetic acid (TCA). The cells were then transferred to cryotubes (Nunc, Roskilde, Denmark), frozen in liquid nitrogen and kept at −80°C. After thawing, samples were centrifuged at 1,000 *g* for 15 min at 4°C. The supernatants were decanted and the pellets resuspended in ice-cold 15% TCA for 20 min. The pellet suspension was then mixed, centrifuged at 1,000 *g* for 15 min at 4°C and pooled with the corresponding supernatants. The TCA supernatants were extracted four times with 5 ml of anhydrous diethylether and adjusted to pH 7.5 with sodium bicarbonate. Sample aliquots (100 μl) were mixed with equal amounts of D-myo-[$^3$H]Ins 1,4,5-$P_3$ and bovine adrenal Ins 1,4,5-$P_3$ binding protein, vortexed and incubated for 15 min on ice. The samples were then centrifugated at 1,000 *g* for 30 min at 4°C. The supernatants were decanted and the pellets were resuspended in scintillant. Radioactivity was then measured by liquid scintillation counting and Ins 1,4,5-$P_3$ determined by comparison with a standard curve containing 0.19, 0.38, 0.76, 1.5, 3.1, 6.2, 12.5 and 25 pmol of Ins 1,4,5-$P_3$. Results are expressed in pmol of Ins 1,4,5-$P_3$ per $10^4$ cells.

*Analysis of inositol phosphates by column chromatography*

To confirm the binding assay, Ins 1,4,5-$P_3$ was measured by fast protein liquid chromatography (FPLC) on a Pharmacia Mono-Q anion-exchange column as described by Chew and Brown (1986) and Bouchelouche et al. (1990). Briefly, for each reaction condition $10^7$ CaKi-1 cells in 75 cm$^2$ flasks were incubated with D-myo-[$^3$H]inositol (5 μCi ml$^{-1}$) for 12 h in an atmosphere of 5% carbon dioxide at 37°C. Cells were then incubated for 30 min with 10 mM cold inositol and 10 mM lithium chloride in McCoy's at 37°C. After incubation cells were washed three times with McCoy's, reincubated with 10 mM lithium chloride in McCoy's for 10 min and stimulated with IFN-γ in 4 ml of McCoy's. The reaction was stopped with 2 ml of ice-cold 15% TCA. The cells were then transferred to cryotubes and TCA supernatants were extracted as described for the radioligand assay measurements of Ins 1,4,5-$P_3$ (see above). Sample aliquots (200 μl) containing the water-soluble inositol phosphates were separated using the Mono-Q anion-exchange column equilibrated with an ammonium formate (M)/formic acid (0.1 M) gradient and an FPLC (Pharmacia) system (Chew & Brown, 1986). Fractions of 3 ml were collected and radioactivities measured by liquid scintillation counting. Three major peaks of radioactivity were eluted from the column. These peaks coeluted with $^3$H-labelled inositol standards (Du Pont/Dupharma, Denmark) of inositol 1-monophosphate (Ins 1-P), inositol 1,4-bisphosphate (Ins 1,4-$P_2$) and Ins 1,4,5-$P_3$ (Chew & Brown, 1986; Bouchelouche et al., 1990).

*Cell viability*

Cell viability was assayed by determining trypan blue exclusion microscopically and scoring a field of approximately 100 cells (Schlager & Adams, 1983).

*Total protein synthesis measurement*

The effect of calcium antagonist and protein inhibitors on total protein synthesis was analysed as described by Ritchie et al. (1991). Briefly, CaKi-1 cells were grown for 48 h in 24-well plates in McCoy's 5a medium containing 2 mM glutamine, 100 IU ml$^{-1}$ penicillin/streptomycin and 10% FBS. Labelling was achieved by incubating cells for 24 h with 20 μCi ml$^{-1}$ [$^{35}$S]methionine with or without test agents. After labelling, the cells were placed on ice and washed twice with ice-cold HBSS. They were lysed in 500 μl of lysis buffer (50 mM Tris pH 8.5/0.5% Triton X-100/0.25% deoxycholate/10 mM EDTA/1 mM phenylmethylsulphonyl fluoride) for 15 min on ice. Lysates were transferred to tubes and a second aliquot of lysis buffer was added to the wells. Lysates were pooled and cleared by a 10 min centrifugation at 4°C. Aliquots of 250 μl were withdrawn from the supernatants to which 250 μl of 20% TCA was added. After a 20 min incubation on ice, the precipitates were transferred to glass microfibre filter discs on a vacuum manifold and washed with 5% cold TCA. Filters were dried and radioactivity was measured by liquid scintillation counting. The means of triplicate experiments are expressed as per cent protein synthesis compared with control cells.

*Flow cytometric analysis of ICAM-1 antigen*

At confluent monolayer in 75 cm$^2$ tissue flasks, CaKi-1 cells were obtained for subculture by addition of 0.15% trypsin (Gibco) in calcium-free phosphate buffer (pH 7.2), blocked by McCoy's 5a with 10% FBS and the detached cells were centrifuged and resuspended in fresh medium. A volume of 1 ml of a $10^7$ cells ml$^{-1}$ suspension was seeded in 25 cm$^2$ tissue flasks (Nunc). After 48 h of culture, cells were treated with IFN-γ, bromo-A23187, diltiazem, EGTA, TMB-8, R24571 and W7 in the indicated combinations, concentrations and time courses. After stimulation, cells were washed twice with HBSS/1% BSA. After washing, cells were detached by incubation in 2 ml of 1 mM EDTA in HBSS/1% BSA for 30 min at 37°C. A 100 μl aliquot of the cell suspension was transferred to 12 × 75 mm polystyrene tubes (Falcon, Becton Dickinson) and incubated for 30 min at 4°C in the dark with fluorescein isothiocyanate (FITC)-conjugated monoclonal anti-ICAM-1, clone 84H10 (Makgoba et al., 1988) (Immunotech, Marseilles, France), diluted 10 μl in 10 μl HBSS. After staining, cells were washed twice with HBSS/1% BSA by centrifigation at 300 *g* for 5 min and resuspended in 500 μl of fixation buffer (HBSS with 1% paraformaldehyde, pH 7.4). Analysis of fluorescence was performed on a FACScan (Becton Dickinson, Mountain View, CA, USA). The background number of fluorescent cells (no relevant monoclonal antibody) was generally adjusted to less than 1% and the relative mean fluorescence intensity of positive cells (MFI) was measured. The irrelevant mouse IgG1 FITC-conjugated antibody X927 (Dako, Denmark) was used as a negative control. All MFI values are presented in a linear scale (Figure 5a–f).

*Assay of protein kinase A activity*

Recent data suggest that cAMP-elevating agents can induce ICAM-1 antigen expression in human glioma cells (Bouillon et al., 1992). In order to evaluate the significance of cAMP-dependent protein kinase (PKA) inhibition by the two modulators of $Ca^{2+}$-binding proteins used in this study, namely R24571 and W7, PKA activity was assayed upon IFN-γ stimulation. CaKi-1 cells were grown to a confluent monolayer in 75 cm$^2$ flask with McCoy's 5a medium supplemented as described above. Following stimulation of the cells ($10^7$) with IFN-γ, treatment was stopped by decanting the medium and rinsing the monolayers twice in ice-cold PBS. After rinsing, flasks were maintained at 4°C while cells were gently scraped in 4 ml of ice-cold extraction buffer (5 mM EDTA, 50 mM Tris, pH 7.5). The cells were homogenised for 15 strokes on ice in a precooled Dounce homogeniser and left on ice for 30 min. To remove cellular debris the homogenate was centrifuged at 2,000 *g* for 10 min. The supernatants were removed and assayed for PKA activity. PKA was measured by the incorporation of $^{32}P$ from 100 μM [γ-$^{32}P$]ATP ($1 \times 10^5$ d.p.m. $nmol^{-1}$, Amersham) into each sample in the presence or absence of cAMP (40 nM) with or without the heat-stable rabbit skeletal muscle PKA inhibitor protein PKI (6–22)$NH_2$ (4 μM in 50 mM Tris, pH 7.5) using the PKA assay system (3128SA, Gibco, Paisley, UK). Substrate phosphorylation in the presence of cAMP reflected the total amount of PKA in each sample. The background (substrate phosphorylation in the presence of PKI) was subtracted from each sample, and PKA activity was expressed as the percentage of activated PKA per total PKA.

### *Statistics*

All results represent the mean ± s.d. of three different experiments. Data were analysed by Student's *t*-test. $P < 0.05$ was considered statistically significant.

### Results

### *Effect of IFN-γ on $[Ca^{2+}]_i$ and possible source of calcium increase*

To determine the effect of IFN-γ on $[Ca^{2+}]_i$ cells were stimulated with 10, 50, 100 and 500 units $ml^{-1}$ IFN-γ and the fura-2/AM-fluorescence was measured continuously with time. Figure 1a shows the dose- and time-dependent change: maximal levels were reached within 1 min of stimulation. In order to examine the ability of IFN-γ to increase $[Ca^{2+}]_i$ by increasing $Ca^{2+}$ influx from the medium as well as by releasing $Ca^{2+}$ from intracellular stores, CaKi-1 cells were incubated with decreasing levels of $Ca^{2+}$ before IFN-γ stimulation. Figure 1b shows the effect of IFN-γ (500 units $ml^{-1}$) in medium containing 1 mM $Ca^{2+}$, 0.32 mM $Ca^{2+}$, or free of $Ca^{2+}$ (omission of calcium and addition of 0.40 mM EGTA). Reducing extracellular $Ca^{2+}$ reduced the IFN-γ-induced rise in $[Ca^{2+}]_i$. In the absence of extracellular $Ca^{2+}$, IFN-γ was still able to induce a $[Ca^{2+}]_i$ response (Figure 1b; Medium: $Ca^{2+}$ free), indicating $Ca^{2+}$ release from intracellular stores. For comparison $[Ca^{2+}]_i$ was measured after stimulation with bromo-A23187, a non-fluorescent calcium ionophore which increases $[Ca^{2+}]_i$ by transporting calcium ions across biological membranes (Luckasen *et al.*, 1974). Figure 1c shows that bromo-A23187 also increased $[Ca^{2+}]_i$ by enhancing $Ca^{2+}$ influx as well as by releasing intracellular $Ca^{2+}$, since there was a significant, but reduced $[Ca^{2+}]_i$ rise in medium free of $Ca^{2+}$. The dependence of the IFN-γ-induced rise in $[Ca^{2+}]_i$ on both $Ca^{2+}$ influx and intracellular $Ca^{2+}$ release was further supported by pretreating CaKi-1 cells with diltiazem (1 μM) or TMB-8 (75 μM) for 60 min before IFN-γ (500 units $ml^{-1}$) stimulation. Figure 1d shows the reduced rise in $[Ca^{2+}]_i$ after blocking $Ca^{2+}$ channels with diltiazem or intracellular $Ca^{2+}$ redistribution with TMB-8 (Malagodi & Chiou, 1974; Owen & Villereal, 1982).

### *Effect of IFN-γ in inositol phosphate formation*

The observed $[Ca^{2+}]_i$ increase to IFN-γ stimulation in $Ca^{2+}$-free medium and reduced response after TMB-8 pretreatment

**Figure 1** Spectrofluoriometric analysis of $[Ca^{2+}]_i$ (nM) in fura-2/AM-loaded CaKi-1 cells, after IFN-γ or bromo-A23187 stimulation. **a**, Effect of IFN-γ stimulation on $[Ca^{2+}]_i$ as related to IFN-γ dose (10, 50, 100 and 500 units $ml^{-1}$). **b**, Effect of IFN-γ (500 units $ml^{-1}$). **c**, Bromo-A23187 (0.5 μM) stimulation of $[Ca^{2+}]_i$ as related to $Ca^{2+}$ in the medium. $Ca^{2+}$-free medium is defined as omission of calcium and addition of 0.40 mM EGTA. **d**, Effect of IFN-γ (500 units $ml^{-1}$) on $[Ca^{2+}]_i$ as related to $Ca^{2+}$ antagonist treatment. Cells were preincubated with TMB-8 (75 μM) or diltiazem (dilt.) (1 μM) for 60 min before IFN-γ stimulation. Control indicates non-pretreated cells. Representative traces of three experiments.

indicates mobilisation of $Ca^{2+}$ from intracellular stores. Therefore, we examined the ability of IFN-γ to increase intracellular levels of Ins 1,4,5-$P_3$ (Table I). Table I shows that IFN-γ caused a concentration-dependent rise in Ins 1,4,5-$P_3$ after 1 min stimulation. IFN-γ at 10 units $ml^{-1}$ was enough for an almost maximal Ins 1,4,5-$P_3$ production. Figure 2 illustrates the time course for Ins 1,4,5-$P_3$ formation after stimulation with 500 units $ml^{-1}$ IFN-γ. Maximal levels were reached within 1 min. This corresponds with the time for maximal $[Ca^{2+}]_i$ increase after stimulation with 500 units $ml^{-1}$ IFN-γ (Figure 1a). In order to confirm the radioligand assay experiments (Table I), inositol phosphate formation was further analysed by FPLC (Table II). As shown in Table II, IFN-γ stimulated a significant increase in Ins 1-P and Ins 1,4,5-$P_3$. There was a slight increase in Ins 1,4,-$P_2$, but this did not reach significant levels. Figure 3 shows a typical elution pattern of water-soluble inositol phosphates from Mono-Q columns. Thus, the FPLC analysis supports the radioligand assay experiments.

### *Effect of calcium antagonists and protein inhibitors on cell viability and total protein synthesis*

To further investigate the role of $Ca^{2+}$ and $Ca^{2+}$-dependent proteins in CaKi-1 cells during IFN-γ stimulation, we tested the effect of diltiazem, EGTA, TMB-8 and $Ca^{2+}$-binding protein inhibitors on IFN-γ-induced ICAM-1 antigen expression. Since ICAM-1 induction may depend on *de novo* protein synthesis, we assayed cell viability and total protein synthesis by trypan blue exclusion and metabolic labelling with [$^{35}$S]methionine to examine non-specific confounding effects of these drugs. Cell viability was unaffected by diltiazem (1 μM), EGTA (1 mM), TMB-8 (75 μM), R24571 (10 μM) or W7 (40 μM) treatment for 24 h. However, the calmodulin antagonist c48/80 (Gietzen, 1983) reduced cell viability to 65–80% with signs of cell detachment at doses as low as 5 μM. In light of these results c48/80 was excluded from the subsequent experiments. Figure 4 shows that, despite normal viability, pretreatment of CaKi-1 cells for 24 h with R24571 (5–10 μM) or W7 (20–40 μM) resulted in a 5–20% decreased protein synthesis. This was not the case for a 24 h pretreatment with diltiazem (1 μM), EGTA (1 mM) or TMB-8 (75 μM). Accordingly, protein synthesis was taken into account for normalising ICAM-1 antigen expression after R24571 and W7 inhibition (Ritchie *et al.*, 1991).

### *Effect of IFN-γ and bromo-A23187 on ICAM-1 antigen expression*

CaKi-1 cells were incubated with 10, 100 or 500 units $ml^{-1}$ IFN-γ for 24 h. The data in Figure 5a show that IFN-γ

**Figure 2** Time-dependent rise in Ins 1,4,5-$P_3$ as determined by radioligand assay in CaKi-1 cells stimulated with 500 units $ml^{-1}$ IFN-γ for 1 min. Values represent mean ± 1 s.d. of three experiments.

**Figure 3** Elution pattern of CaKi-1 cell [$^3$H]inositol phosphates isolated with a Pharmacia Mono-Q column. Open circles indicate basal values and closed circles indicate values from IFN-γ (500 units $ml^{-1}$ for 1 min)-stimulated cultures. Representative trace of three experiments.

**Table I** Radioimmunoassay measurement of Ins 1,4,5-$P_3$ in CaKi-1 cells after 1 min stimulation with IFN-γ (mean ± s.d. of three experiments)

| *IFN-γ (units $ml^{-1}$)* | *Ins 1,4,5-$P_3$ (pmol $10^{-4}$ cells)* |
|---|---|
| 0 (control) | 0.3 ± 0.1 |
| 10 | 1.1 ± 0.1 ($P$ <0.02) |
| 100 | 1.2 ± 0.4 ($P$ <0.02) |
| 500 | 1.6 ± 0.3 ($P$ <0.02) |

**Figure 4** Effect of calcium antagonists and protein inhibitors on total protein synthesis. CaKi-1 cells were treated for 24 h with diltiazem (dilt.), EGTA, TMB-8, R24571 or W7 in the indicated concentrations. Protein synthesis was assessed by [$^{35}$S]methionine incorporation. Data represent mean ± 1 s.d. of three experiments. *Significant differences from control value.

**Table II** FPLC analysis of inositol phosphate turnover in CaKi-1 cells after 1 min stimulation with IFN-γ (500 units $ml^{-1}$) (mean ± s.d. of three experiments)

| *Inositol phosphates (radioactivity, c.p.m. per fraction)* | *Control* | *IFN-γ* |
|---|---|---|
| Ins 1-P | 193 ± 10 | 467 ± 7 ($P$ <0.01) |
| Ins 1,4-$P_2$ | 35 ± 6 | 84 ± 35 (non-significant) |
| Ins 1,4,5-$P_3$ | 35 ± 12 | 129 ± 9 ($P$ <0.01) |

**Figure 5** FACS analysis of ICAM-1 antigen expression in CaKi-1 cells. **a**, Cells were incubated with increasing doses of IFN-γ for 24 h. **b**, Effect of 24 h incubation with bromo-A23187 (0.5 μM) and/or IFN-γ (500 units $ml^{-1}$). **c**, Effect of diltiazem (dilt.) (1 μM), EGTA (1 mM) or **d**, TMB-8 (75 μM) on IFN-γ (500 units $ml^{-1}$)-induced ICAM-1 antigen expression. Cells were pretreated with inhibitors for 60 min before IFN-γ incubation for 24 h. **e**, Effect of R24571 (10 μM) or **f**, W7 (40 μM) on IFN-γ (500 units $ml^{-1}$) induction. Cells were pretreated with inhibitors for 60 min before IFN-γ incubation for 24 h. ICAM-1 (MFI) was normalised according to total protein synthesis for R24571 (R24571-Normalised) and W7 (W7-Normalised). Data represent mean ± 1 s.d. of three experiments. *, **Significantly ($P<0.05$) higher than control and IFN-γ values respectively. ***Significantly ($P<0.05$) lower than IFN-γ value.

caused an increase in ICAM-1 antigen expression above control value at 100 and 500 units $ml^{-1}$. IFN-γ at 10 units $ml^{-1}$ had no effect on ICAM-1 antigen expression, although this dose yielded a clear $[Ca^{2+}]_i$ rise (Figure 1a) and an almost maximal Ins 1,4,5-$P_3$ production (Table I). To investigate the role of calcium during ICAM-1 antigen induction we incubated cells with bromo-A23187 (0.5 μM) for 24 h. Bromo-A23187 significantly raised ICAM-1 antigen expression after 24 h (Figure 5b). Furthermore, combining full doses of IFN-γ (500 units $ml^{-1}$) and bromo-A23187 for 24 h significantly increased ICAM-1 antigen expression as compared with either agent alone (Figure 5b). This increment showed a slight synergy with a mean MFI of 272, since the sum of the two agents mean increment was 187 MFI.

### *Effects of inhibition of calcium transients on IFN-γ-induced ICAM-1 antigen expression*

To determine the functional significance of extracellular calcium, cells were pretreated for 60 min at 37°C with the calcium channel blocker diltiazem (1 μM) or the $Ca^{2+}$ chelator EGTA (1 mM), before addition of IFN-γ (500 units $ml^{-1}$) for 24 h. Figure 5c shows that, in the presence of diltiazem or EGTA, the mean MFI decreased from 531 ± 5

to 508 ± 7 and 493 ± 23 respectively. Despite statistical significance, these differences represent a weak non-conclusive decline in ICAM-1 antigen expression. The two antagonists had no effect on basal ICAM-1 antigen expression when used alone. To examine whether cellular calcium stores contribute to an increase in ICAM-1 antigen expression, CaKi-1 cells were pretreated with TMB-8 (75 μM) for 60 min at 37°C followed by addition of IFN-γ (500 units $ml^{-1}$) for 24 h. TMB-8 significantly reduced IFN-γ-induced ICAM-1 antigen expression from 579 ± 1 (MFI) to 514 ± 1 (MFI). This represents a partial reduction of only 11%. TMB-8 had no effect on basal ICAM-1 antigen expression.

*Effect of R24571 and W7 on IFN-γ-induced ICAM-1 antigen expression*

Since ICAM-1 antigen expression may be induced by a $[Ca^{2+}]_i$ rise via activation of $Ca^{2+}$-binding proteins, CaKi-1 cells were pretreated with R24571, an anti-mycotic agent, and W7, a naphthalenesulphonamide, both potent inhibitors of $Ca^{2+}$-binding proteins including PKC and calmodulin (Gietzen *et al.*, 1981; Tanaka *et al.*, 1983). Incubation of cells with R24571 (10 μM) or W7 (40 μM) for 60 min at 37°C before addition of IFN-γ (500 units $ml^{-1}$) for 24 h significantly reduced IFN-γ-induced ICAM-1 antigen expression (Figure 5e and f). However, when the total protein synthesis was taken into account for normalising ICAM-1 antigen expression, R24571 had no inhibitory effect, while W7 only inhibited ICAM-1 antigen induction from 579 ± 1 (MFI) to 526 ± 22 (MFI), less than 10% (Figure 5e: IFN-γ + R24571-Normalised; and Figure 5f: IFN-γ + W7-Normalised). Since the differences in ICAM-1 antigen expression obtained upon IFN-γ treatment with or without calcium antagonists or protein inhibitors were weak, we examined the FACS profile (cell distribution) in addition to the MFI. Figure 6 illustrates representative traces from the FACS analysis indicating a highly homogeneous CaKi-1 cell population with minimal variation.

*Effect of IFN-γ on PKA activity*

The control level of PKA activity in CaKi-1 cells was 6.0 ± 1.7%. This level was unaffected by stimulation with IFN-γ (500 units $ml^{-1}$) for 15, 30 and 60 min, which yielded a PKA activity between 5.6 ± 1.4% and 7.8 ± 1.5%. These results support the hypothesis that the inhibitory effect of W7 on IFN-γ-induced ICAM-1 antigen expression was not caused by PKA inhibition. Hence, although cAMP-elevating agents can enhance ICAM-1 antigen expression, possibly via PKA (Bouillon *et al.*, 1992), and although the potent calmodulin antagonist W7 may act upon PKA at 40 μM (Hidaka *et al.*, 1984), this was unlikely to be the case in CaKi-1 cells.

**Figure 6** FACS profile from the analysis of ICAM-1 antigen expression on CaKi-1 cells. Cells were untreated (control) or treated with IFN-γ (500 units $ml^{-1}$) for 24 h with or without a 60 min preincubation with TMB-8 (75 μM) (top) or W7 (40 μM) (bottom).

### Discussion

IFN-γ has been used alone or in combination with IFN-α as an immunotherapeutic agent in patients with renal cell carcinoma (Heicappell & Ackermann, 1990). One of the main anti-tumoral effects of IFN-γ is the induction of cell-surface antigens, including ICAM-1, by activating different intracellular signal transduction pathways (Azuma *et al.*, 1992; Bouillon *et al.*, 1992; Hansen *et al.*, 1993). In different cell types IFN-γ has been shown to activate PKC and/or increase Ins 1,4,5-$P_3$ formation, indicating stimulation of the phosphatidylinositol-$Ca^{2+}$ signal transduction pathway (Klein *et al.*, 1990; Renkonen *et al.*, 1990). We have recently demonstrated PKC activation as well as ICAM-1 antigen induction upon IFN-γ treatment of CaKi-1 cells (Hansen *et al.*, 1993). Therefore, for the first time, we decided to examine the role of $Ca^{2+}$ and Ins 1,4,5-$P_3$ formation during IFN-γ stimulation of this human renal carcinoma cell line, and relate these findings to ICAM-1 antigen induction.

Our findings showed a dose-dependent ability of IFN-γ to increase $[Ca^{2+}]_i$ by inducing influx of $Ca^{2+}$ and redistribution of intracellular $Ca^{2+}$ stores. An influx of extracellular calcium after IFN-γ stimulation was supported by the inhibition of the rise in $[Ca^{2+}]_i$ by removal of extracellular $Ca^{2+}$ (Figure 1b) and by blocking calcium channels with diltiazem (Figure 1d). Redistribution of intracellular calcium was supported by the failure to completely block the increase in $[Ca^{2+}]_i$ with $Ca^{2+}$-free medium and by inhibition with TMB-8 (Figure 1d). Since TMB-8 is a known inhibitor of intracellular $Ca^{2+}$ redistribution (Malagodi & Chiou, 1974; Owen & Villereal, 1982), and since Ins 1,4,5-$P_3$ is a natural releaser of $Ca^{2+}$ from intracellular stores (Downes & Macphee, 1990), IFN-γ-stimulated formation of Ins 1,4,5-$P_3$ was examined. We found a signficant increment in Ins 1,4,5-$P_3$ using two independent methods, a radioligand assay (Table I and Figure 2) and FPLC (Table II and Figure 3), which suggests that IFN-γ-induced redistribution of intracellular calcium may be mediated by this phosphatidylinositol metabolite. Usually high-performance liquid chromatography (HPLC) is used for separation of inositol phosphates (Burgess *et al.*, 1985; Turk *et al.*, 1986; Klein *et al.*, 1990). However, the FPLC system appears to be superior in its ability to rapidly separate inositol phosphates while maintaining good resolution (Florholmen *et al.*, 1989).

Often, only cell viability by dye exclusion or 'signs of toxicity' not otherwise specified are described when protein inhibitors are used in the study of antigen expression in cell cultures (Rothlein *et al.*, 1988; Griffiths *et al.*, 1990; Pedrinaci

*et al.*, 1990). However, this may be misleading, since total protein synthesis can be inhibited despite normal cell viability, as shown for R24571 and W7 in CaKi-1 cells (Figure 4). The inhibition of total protein synthesis by these two inhibitors is a non-specific effect in relation to ICAM-1 antigen expression. Normalising ICAM-1 antigen expression to total protein synthesis revealed a markedly reduced effect, with only W7 reaching significant inhibition (Figure 5e and f). Similar findings have been obtained with the PKC inhibitors H7 and staurosporine (Ritchie *et al.*, 1991; Bouillon & Audette, 1993). Unfortunately, compound 48/80 could not be used because the cell viability was affected at even low doses.

The enhancement of ICAM-1 antigen expression in CaKi-1 cells by IFN-γ as assessed by FACS was approximately 1.3- to 1.4-fold. Although this represents a weak response as compared with other cell types, such as melanoma cells (Scheibenbogen *et al.*, 1993), the FACS profile showed a highly homogeneous cell population (Figure 6). Furthermore, our data clearly demonstrated that IFN-γ-induced Ins 1,4,5-$P_3$ formation was not coupled to ICAM-1 antigen expression because 10 units $ml^{-1}$ IFN-γ was enough for an almost maximal Ins 1,4,5-$P_3$ formation (Table I), whereas it had no effect on ICAM-1 antigen expression (Figure 5a). Also, in the presence of either diltiazem, EGTA or TMB-8, the IFN-γ-stimulated ICAM-1 antigen expression decreased by less than 15% (Figure 5c and d). Therefore, although statistically significant, such weak differences may not reflect a biochemically relevant phenomenon. Hence, the lack of coupling between Ins 1,4,5-$P_3$ formation and ICAM-1 antigen induction by IFN-γ as well as the lack of inhibition of this induction by calcium antagonists indicate that a $Ca^{2+}$-phophatidylinositol signalling mechanism does not play a major role in IFN-γ-induced ICAM-1 antigen expression in CaKi-1 cells. This does not exclude the possibility that raising $[Ca^{2+}]_i$ by mechanisms other than those applied by IFN-γ can potentiate IFN-γ induction. Thus, as illustrated in Figure 5b, raising $[Ca^{2+}]_i$ with bromo-A23187 synergistically enhanced IFN-γ-induced ICAM-1 antigen expression. If IFN-γ raised ICAM-1 antigen expression by increasing $[Ca^{2+}]_i$, then its combined effect with bromo-A23187 may have been predicted to be additive. The minor role of a $Ca^{2+}$-dependent mechanism during ICAM-1 antigen induction by IFN-γ was further supported by the weak inhibition of this induction by W7 (Figure 5f). The naphthalenesulphonamide derivative W7 is a potent calmodulin antagonist, but may act upon several protein kinases at high concentrations (Tanaka *et al.*, 1983: Hidaka *et al.*,1984). These include the cAMP-dependent PKA and PKC, besides its actions via calmodulin on calmodulin-dependent proteins. Since both cAMP-elevating agents and PKC activators can enhance ICAM-1 antigen expression (Griffiths *et al.*, 1990; Bouillon *et al.*, 1992) the W7 effect could have been ascribed to PKA and/or PKC inhibition, and not just calmodulin antagonism. However, IFN-γ did not stimulate PKA activity in CaKi-1 cells. Furthermore, although IFN-γ activates PKC in CaKi-1 cells as assayed by both histone and acetylated myelin basic protein peptide (4-14) substrate phosphorylation, inhibition of PKC with H7 and sphingosine had no effect on IFN-γ-induced ICAM-1 antigen expression (Hansen *et al.*, 1993). In light of these findings, it is very unlikely that W7, a weaker PKC inhibitor than H7 and sphingosine, abrogated that IFN-γ induction by affecting PKA or PKC. The exact calmodulin-dependent proteins affected by W7 in our model are unknown, since calmodulin can activate a wide range of kinases and cyclases, including CaM kinase II, phosphorylase kinase, adenylate cyclase and guanylate cyclase (Brady *et al.*, 1985). As discussed above, the non-specific effect of W7 on total protein synthesis was accounted for by normalising ICAM-1 antigen expression.

Our results partly agree with earlier studies on IFN-γ signalling in other cell types. In human endothelial cells, IFN-γ activates PKC and induces Ins 1,4,5-$P_3$ (Renkonen *et al.*, 1990). Using similar radioligand assay conditions for Ins 1,4,5-$P_3$ quantitation as in our study, Renkonen *et al.* (1990) showed a maximal rise after 10 min of stimulation, with no significant rise within 1 min. This discrepancy, as compared with CaKi-1 cells, may relate to the malignant transformation itself or to the different embryonal origins of the cell lines. However, Renkonen *et al.* (1990) did not confirm their measurements of inositol trisphosphate formation with other independent assays, but were able to show a significant 1.4-fold increase in the efflux of $^{45}Ca^{2+}$ 5 min after IFN-γ stimulation. Furthermore, although W7 was shown to decrease IFN-γ-induced ICAM-1 antigen expression, this decrease was less than 20% and also not normalised to total protein synthesis. Therefore, the W7 effect may well have been non-specific. Our data are consistent with the findings of Klein *et al.* (1990), who demonstrated that IFN-γ caused a significant increase in Ins 1,4,5-$P_3$ formation within 1 min in U937 cells, using a identical radioligand assay and HPLC. The IFN-γ-induced rise in Ins 1,4,5-$P_3$ was correlated to a rise in $[Ca^{2+}]_i$. In agreement with our study, W7 had little or no effect on IFN-γ-induced antigen expression in U937 cells. In contrast, Ina *et al.* (1987) have shown that IFN-γ induces a rise in $Ca^{2+}$ and HLA class II antigen expression in HL-60 cells, which again can be efficiently blocked by W7. In general, the reports on IFN-γ signalling agree that this cytokine can activate PKC and raise $[Ca^{2+}]_i$ by increasing $Ca^{2+}$ influx and/or by generating Ins 1,4,5-$P_3$. However, the effect of PKC inhibitors such as H7 or calmodulin inhibitors such as W7 on IFN-γ-induced antigen expression depends on the applied cell line and antigen under study (Ina *et al.*, 1987; Griffiths *et al.*, 1990; Klein *et al.*, 1990; Renkonen *et al.*, 1990; Hansen *et al.*, 1993).

We conclude that IFN-γ can induce a rise in $[Ca^{2+}]_i$ in CaKi-1 cells which is dependent upon $Ca^{2+}$ influx as well as release of $Ca^{2+}$ from intracellular stores, probably as a result of Ins 1,4,5-$P_3$ generation. This $Ca^{2+}$-Ins 1,4,5-$P_3$ response plays only a minor role during IFN-γ-induced ICAM-1 antigen expression and further studies are needed to identify other signal transduction pathways for IFN-γ in our model.

The authors wish to thank Annie Olsen and Lisbet von Kappelgaard for their skilled technical and secretarial assistance. This study was supported by the Director Jacob Madsen and wife Olga Madsen Foundation (A.B.H.), The Danish Hospital Foundation for Medical Research, Region of Copenhagen, The Faroe Islands and Greenland (A.B.H.), Torben Linnemann's grant for cancer research (A.B.H.) and the Danish Cancer Society (P.N.B.).

### References

AZUMA, A., YAGITA, H., MATSUDA, H., OKUMURA, K. & NIITANI, H. (1992). Induction of intercellular adhesion molecule 1 on small cell lung carcinoma cell lines by γ-interferon enhances spontaneous and bispecific Anti-CD3 X Antitumor antibody-directed lymphokine activated killer cell cytotoxicity. *Cancer Res.*, **52**, 4890-4894.

BOUCHELOUCHE, P.N., REIMERT, C. & BENDTZEN, K. (1988). Effects of natural and recombinant interleukin 1α and -β on cytosolic free calcium in human and murine fibroblasts. *Leukemia*, **2**, 691-696.

BOUCHELOUCHE, P.N., AHNFELT-RØNNE, I. & THOMSEN, M.K. (1990). $LTD_4$ increases cytosolic free calcium and inositol phosphates in human neutrophils: inhibition by the novel $LTD_4$ receptor antagonist, SR2640, and possible relation to modulation of chemotaxis. *Agents Actions*, **29**, 299-307.

BOUILLON, M. & AUDETTE, M. (1993). Transduction of Retinoic acid and γ-interferon signal for intercellular adhesion molecule-1 expression in human tumor cell lines: Evidence for the late-acting involvement of protein kinase C inactivation. *Cancer Res.*, **53**, 826-832.

BOUILLON, M., FORTIER, M.A., BOULIANNE, R. & AUDETTE, M. (1992). Biphasic effect of cAMP-elevating agents on ICAM-1 expression stimulated by retinoic acid and interferon-γ. *Int. J. Cancer*, **50**, 281–288.

BRADY, R.C., CABRAL, F.R., SCHIBLER, M.J. & DEDMAN, J.R. (1985). Cellular localization of calmodulin and calmodulin-acceptor protens. In *Calcium and Cell Physiology*, Marme, D. (ed.), pp. 140–169. Springer: Berlin.

BURGESS, G.M., MCKINNEY, J.S., IRVINE, R.F. & PUTNEY, J.W. (1985). Inositol 1,4,5-trisphosphate and inositol 1,3,4-trisphosphate formation in $Ca^{2+}$ mobilizing hormone activated cells. *Biochem. J.*, **232**, 237–243.

CHALISS, R.A.J., BATLY, I.H. & NAHORSKI, S.R. (1988). Mass measurement of inositol (1,4,5)trisphosphate in rat cerebral cortex slices using a radioreceptor assay: effect of neurotransmitters and depolarization. *Biochem. Biophys. Res. Commun.*, **157**, 684–691.

CHEW, C.S. & BROWN, M.R. (1986). Release of intracellular calcium and elevation of inositol trisphosphate by secretagogues in parietal and chief cells isolated from rabbit gastric mucosa. *Biochim. Biophys. Acta*, **888**, 116–125.

DOWNES, P.C. & MACPHEE, C.H. (1990). Review: myo-inositol metabolites as cellular signals. *Eur. J. Biochem.*, **193**, 1–18.

FAN, X.-D., GOLDBERG, M. & BLOOM, B.R. (1988). Interferon-γ-induced transcriptional activation is mediated by protein kinase C. *Proc. Natl Acad. Sci. USA*, **85**, 5122–5125.

FLORHOLMEN, J., MALM, D., VONEN, B. & HURHOL, P.G. (1989). Effect of cholecystokinin on the accumulation of inositol phosphates in isolated pancreatic islets. *Am. J. Phys.*, **257**, G865–G870.

FOGH, J. & TREMPE, G. (1975). *Human Tumor Cells* in vitro, pp. 115–159. Plenum: New York.

GIETZEN, K. (1983). Comparison of the calmodulin antagonists compound 48/80 and calmidazolium. *Biochem. J.*, **216**, 611–616.

GIETZEN, K., WÜTHRICH, A. & BADER, H. (1981). R24571: a new powerful inhibitor of red blood cell $Ca^{2+}$-transport ATP'ase and of calmodulin-regulated functions. *Biochem. Biophys. Res. Commun.*, **101**, 418–425.

GRIFFITHS, C.E.M., ESMANN, J., FISHER, G.J., VOORHEES, J.J. & NICKOLOFF, B.J. (1990). Differential modulation of keratinocyte intercellular adhesion molecule-1 expression by γ interferon and phorbol ester: evidence for involvement of protein kinase C signal transduction. *Br. J. Dermatol.*, **122**, 333–342.

HANSEN, A.B., BOUCHELOUCHE, P.N., GIESE, B.N. & ANDERSEN, C.B. (1993). Role of protein kinase C during interferon-γ and phorbol ester stimulated immunocytochemical expression of ICAM-1 in human renal carcinoma cells. *Acta Pathol. Microbiol. Immunol. Scand.*, **101**, 437–448.

HEICAPPELL, R. & ACKERMANN, R. (1990). Rationale for immunotherapy of renal cell carcinoma. *Urol. Res.*, **18**, 357–372.

HIDAKA, H., INAGAKI, M., KAWAMOTO, S. & SASAKI, Y. (1984). Isoquinolinesulfonamides, novel and potent inhibitors of cyclic nucleotide dependent protein kinase and protein kinase C. *Biochemistry*, **23**, 5036–5041.

INA, Y., KOIDE, Y., NEZU, N. & YOSHIDA, T.O. (1987). Regulation of HLA class II antigen expression: intracellular signalling molecules responsible for the regulation by IFN-γ and cross-linking of Fc receptors in HL-60 cells. *J. Immunol.*, **193**, 1711–1717.

KLEIN, J.B., SCHEPERS, T.M., DEAN, W.L., SONNENFELD, G. & MCLEISH, K.R. (1990). Role of intracellular calcium concentration and protein kinase C activation in IFN-γ stimulation of U937 cells. *J. Immunol.*, **144**, 4305–4311.

LUCKASEN, J.R., WHITE, J.G. & BEISEY, H. (1974). Mitogenic properties of a calcium ionophore, A23187. *Proc. Natl Acad. Sci. USA*, **71**, 5088–5090.

MAKGOBA, M.W., SANDERS, M.E., GINTER LUCE, T.E., DUSTIN, M.L., SPRINGER, T.A., CLARK, E.A., MANNONI, P. & SHAW, S. (1988). ICAM-1: a ligand for LFA-1 dependent adhesion of B, T and myeloid cells. *Nature*, **331**, 86–88.

MALAGODI, M.H. & CHIOU, C.Y. (1974). Pharmacological evaluation of a new $Ca^{++}$ antagonist, 8-(N,N-diethylamino)-octyl-3,4,5-trimethoxybenzoate hydrochloride (TMB-8): studies in smooth muscles. *Eur. J. Pharmacol.*, **27**, 25–33.

MORTARINI, R., BELLI, F., PARMIANI, G. & ANICHINI, A. (1990). Cytokine-mediated modulation of HLA-class II, ICAM-1, LFA-3 and tumor-associated antigen profile of melanoma cells. Comparison with anti-proliferative activity by rIL1-β, rTNF-α, rIFN-γ, rIL4 and their combinations. *Int. J. Cancer*, **45**, 334–341.

OWEN, N.E. & VILLEREAL, M.L. (1982). Effect of the intracellular $Ca^{++}$ antagonist TMB-8 on serum stimulated $Na^+$ influx in human fibroblasts. *Biochem. Biophys. Res. Commun.*, **109**, 762–768.

PALMER, S., HUGHES, K.T., LEE, D.Y. & WAKELAM, M.J.O. (1989). Development of a novel, INS(1,4,5)$P_3$-specific binding assay. Its use to determine the intracellular concentration of INS(1,4,5)$P_3$ in unstimulated and vassopressin stimulated rat hepatocytes. *Cell. Signalling*, **1**, 147–156.

PEDRINACI, S., RUIZ-CABELLO, F., GOMEZ, O., COLLADO, A. & GARRIDO, F. (1990). Protein kinase C-mediated regulation of the expression of CD14 and CD11/CD18 in U937 cells. *Int. J. Cancer*, **45**, 294–298.

RENKONEN, R., MENNANDER, A., USTINOV, J. & MATTILA, P. (1990). Activation of protein kinase C is crucial in the regulation of ICAM-1 expression on endothelial cells by interferon-γ. *Int. Immunol.*, **2**, 719–729.

RITCHIE, A.J., JOHNSON, D.R., EWENSTEIN, B.M. & POBER, J.S. (1991). Tumor necrosis factor induction of endothelial cell surface antigens is independent of protein kinase C activation or inactivation. Studies with phorbol myristate acetate and staurosporine. *J. Immunol.*, **146**, 3056–3062.

ROTHLEIN, R., CZAJKOWSKI, M., O'NEIL, M.M., MARLIN, S.D., MAINOLFI, E. & MERLUZZI, V.J. (1988). Induction of intercellular adhesion molecule 1 on primary and continuous cell lines by pro-inflammatory cytokines. Regulation by pharmacologic agents and neutralizing antibodies. *J. Immunol.*, **141**, 1665–1669.

SCHEIBENBOGEN, C., KEILHOLZ, U., MEUER, S., DENGLAR, T., TILGEN, W. & HUNSTEIN, W. (1993). Differential expression and release of LFA-3 and ICAM-1 in human melanoma cell lines. *Int. J. Cancer*, **54**, 494–498.

SCHLAGER, S.I. & ADAMS, A.C. (1983). Use of dyes and radioisotopic markers in cytotoxicity test. *Methods Enzymol.*, **93**, 233–245.

TANAKA, Y., YAMADA, E., SONE, T. & HIDAKA, H. (1983). Calcium-independent activation of calcium ion-dependent cyclic nucleotide phosphodiesterase by synthetic compounds: quinazolinesulfonamide derivates. *Biochemistry*, **22**, 1030–1034.

TURK, J., WOLF, B.A. & MCDANIEL, M.L. (1986). Glucose induced accumulation of inositol trisphosphates in isolated pancreatic islets. *Biochem. J.*, **237**, 259–263.